# The positive impact of identity-affirming mental health treatment for neurodivergent individuals

**DOI:** 10.3389/fpsyg.2024.1403129

**Published:** 2024-07-15

**Authors:** Elizabeth Kroll, Megan Lederman, Jonathan Kohlmeier, Komal Kumar, Jaime Ballard, Izabella Zant, Caroline Fenkel

**Affiliations:** ^1^Charlie Health, Inc., Bozeman, MT, United States; ^2^The Center for Applied Research and Educational Improvement, University of Minnesota, Saint Paul, MN, United States

**Keywords:** neurodivergence, identity, mental health, IOP, treatment, affirming care, Intensive Outpatient Program

## Abstract

**Introduction:**

The medical and social definitions of neurodivergence have become a common topic of discussion in recent years, and the ways that we define, measure and report on conditions within the neurodivergent umbrella are changing. The objective of this study was to analyze differences in mental health symptom presentation at intake and compare treatment outcomes among three groups: clients with an affirming neurodivergent diagnosis, clients without an affirming diagnosis, and neurotypical clients.

**Methods:**

Data were collected at intake and discharge. Clients self-reported neurodivergent identity, neurodivergent diagnoses, as well as the severity of depression symptoms, anxiety symptoms and self-harm frequency. One-way multivariate analysis of variance tests were run to assess differences in mental health symptoms at intake and discharge based on neurodivergent identity and corresponding diagnosis. When MANOVAs indicated significant differences, follow-up univariate one-way ANOVAs were conducted for each dependent variable.

**Results:**

Neurodivergent clients reported significantly worse mental health symptoms at intake than neurotypical clients, regardless of diagnosis status. Additionally, clients who identified as neurodivergent but did not report an affirming medical diagnosis reported significantly worse mental health symptoms than those who did report an affirming medical diagnosis. By discharge from IOP treatment, no significant differences were found in symptom change scores between neurodivergent and neurotypical individuals, or neurodivergent individuals with an affirming diagnosis and those without.

**Discussion:**

These findings highlight the importance of acknowledging client identity as a key component of mental health treatment. The act of validating symptoms and experiences, allowing accommodations when requested, and exploring identity formation regardless of diagnosis, allowed all clients who identified as neurodivergent to benefit from treatment.

## 1 Introduction

### 1.1 What is neurodivergence?

As a relatively new concept, neurodivergence seems to have multiple definitions and no consensus on which definition to use. It’s both a collection of medical conditions (American Psychiatric Association, 2013), and an identity (Lewis et al., 2016; Lewis et al., 2017). It can be a set of symptoms that impact daily functioning (Pellicano and den Houting, 2022), but also a different way of processing information (Singer, 1999; Jaarsma and Welin, 2012). To simplify this concept, neurodivergence is often viewed through two lenses: the medical model, and the social model. While other models of neurodivergence do exist (Dwyer, 2022), in this paper, we focus on exploring and comparing symptoms and outcomes between clients whose neurodiversity is affirmed or denied by the social and medical models specifically, so as not to overcomplicate the analysis.

Medically, neurodivergence is equated to neurodevelopmental disabilities. These are defined by the *Diagnostic and Statistical Manual of Mental Disorders* (DSM-5) as “a group of conditions with onset in the developmental period. The disorders typically manifest early in development, often before the child enters grade school, and are characterized by developmental deficits that produce impairments of the personal, social, academic, or occupational functioning.” (American Psychiatric Association, 2013, p 31). These disorders include intellectual disorders, communication disorders, Autism Spectrum Disorder (ASD), Attention-Deficit/Hyperactivity Disorder (ADHD), Specific Learning disorders, motor disorders, and Tic disorders. Neurodevelopmental disorders are often summarized as any condition in which someone develops atypical cognitive processes early in life. Critics of the medical model argue that it pathologizes normally occurring variations in processing and can therefore lead to stigmatization and marginalization of neurodivergent individuals. Often, the goal of treatment in the medical model is seen as “normalization” or “curing” of neurodivergent individuals.

From a social perspective, the concept of neurodivergence embodies a broader and more inclusive definition than the medical model. For some, it has come to mean anything that resembles processing, learning, or behaving differently from what is considered normal or typical, including common neurodevelopmental disorders such as attention deficit hyperactive disorder (ADHD), and non-developmental disorders such as anxiety or OCD. Neurodiversity, established as a concept within online autistic communities in the mid 1990s (Botha et al., 2024), and further expounded upon in the late 1990s by journalist Blume (1998) and sociologist Singer (1999), is equated to biodiversity: if biodiversity makes an ecosystem stronger, then neurodiversity may do the same for culture and society. It has become a popular social topic over the past few years, with Google Trends-a (2023a) and Google Trends-b (2023b) showing spikes in searches for the words “neurodivergent” and “neurodivergence” starting in April of 2020. The neurodiversity paradigm follows the disability movement, claiming that the struggles that neurodivergent individuals face are not a result of the symptoms that they present, but rather the rigidity of the environment.

Historically, the medical model excluded neurodivergent viewpoints (Milton, 2014), as neurodivergent individuals were seen as less intelligent and less reliable than their neurotypical counterparts (Chellappa, 2023). However, epistemology suggests that neurodivergent individuals have a deeper knowledge and understanding of the neurodivergent experience than non-neurodivergent individuals, including professionals. Simply put, the neurodivergent experience is best understood by neurodivergent individuals themselves. A 2017 study surveyed 636 adults and found that autistic individuals tended to have a less biased and more scientific understanding of autism than non-autistic respondents (Gillespie-Lynch et al., 2017), thus supporting the argument that neurodivergent individuals should be included in the foundational research around what it means to be neurodivergent.

In an attempt to reframe neurodivergence within the research community and society at large, advocates emphasize the importance of participatory research with the neurodivergent community (Pellicano and den Houting, 2022; Sonuga-Barke et al., 2023). As neurodivergent voices are included in the growing research and treatment of conditions falling under the neurodevelopmental umbrella, beliefs around what constitutes neurodivergence and what it means to be neurodivergent are beginning to change and the language that we use to describe neurodivergence is changing as well. Throughout the remainder of this paper, identity-first language will be used when describing neurodivergent individuals, as a large portion of neurodivergent individuals prefer this model compared to person-first language (Chapman and Bovell, 2022; Wooldridge, 2023). Furthermore, we acknowledge that the neurodivergent community prefers the social model over the medical model when describing neurodivergence and emphasize that medical language is only used throughout the paper when necessary to illustrate the differences between the two theories.

### 1.2 Medical diagnosis vs. self-identification

It is estimated that approximately 20% of the population falls within the medical definition of “neurodivergence” (Doyle, 2020). Of the neurodevelopmental diagnoses listed previously, ASD and ADHD are the most well known and most commonly diagnosed, with 2.7% of children between the ages of 3–17 receiving an ASD diagnosis, and 9.8% receiving an ADHD diagnosis (Centers for Disease Control and Prevention, 2023a,b). Furthermore, both ASD and ADHD have seen a recent increase in diagnoses, with a 400% increase in ASD diagnoses between 2000 and 2020 (Maenner et al., 2023), and up to a 200% increase in ADHD diagnoses between 2005 and 2014 (Davidovitch et al., 2017). However, it is still debated to what degree these increases are due to an increase in occurrence, a greater understanding of the disorder, or an overdiagnosis.

Due to the growing social movement around neurodiversity and rising calls for neurodivergent self-advocacy, self-identifying as neurodivergent is becoming more common (Lewis, 2016; Lewis, 2017), regardless of whether a person has a medical diagnosis or not. Self-identification is seen by some as an important step in identity formation for neurodivergent individuals (Wylie, 2014). Additionally, research has shown that diagnosed autistic individuals and self-identified autistic individuals have similar autism identity scores, as well as rates of internal stigma, quality of life, and self-esteem, all of which are significantly worse than the general population (McDonald, 2020). The results from this study indicate a strong inherent similarity between medically diagnosed individuals and self-identifying individuals and calls for additional investigation into the validity of self-identification.

There are a multitude of reasons why neurodivergent individuals may be self-identifying and not medically diagnosed. Receiving a diagnosis for many of the medically defined neurodivergent conditions can be difficult. Common barriers include access, cost, and fear of not being believed (Lewis, 2017). As an additional barrier, neurodivergent individuals who did not receive a diagnosis as a child have a difficult time finding providers who have experience evaluating and treating adults, leading to further delays in receiving proper diagnoses (Jones et al., 2014). All of these barriers delay and ultimately discourage neurodivergent individuals from seeking a diagnosis at all and, as a result, they remain only self-identified as neurodivergent instead of formally diagnosed. Furthermore, some neurodivergent individuals do not feel the need for a medical diagnosis, claiming that they receive the support and resources they need without a diagnosis.

However, lack of diagnosis has been correlated with increased mental health issues such as loneliness, isolation, underachievement at school, general unhappiness, and anxiety (Mason et al., 2023). Conversely, receiving a medical diagnosis for a neurodivergent disorder may have benefits. Earlier diagnosis has been shown to improve family support and has been noted as an important gateway for parent understanding and adaptation with neurodivergent children (Vanaken et al., 2023), and diagnosis at any age is associated with increased self-acceptance (Moore, 2016; Hickey et al., 2018; Lilley et al., 2022).

### 1.3 Co-occuring conditions

Medically diagnosed neurodivergent individuals report a high rate of co-occurring psychiatric disorders, such as depression, anxiety, conduct disorder, and substance dependence (Biederman et al., 1999, 2006; Gupte-Singh et al., 2017; Riglin et al., 2021). ADHD and depression co-occur anywhere between 35 and 50% of the time (Gnanavel et al., 2019) and this co-occurence is associated with a higher rate of suicidality, and a higher likelihood of needing psychiatric hospitalization (Biederman et al., 2008). ADHD youth are 4–6 times more likely to develop depression than non-ADHD youth, and ADHD girls with depression have a significantly higher risk of suicidal ideation than ADHD boys diagnosed with depression (Kessler et al., 2006; Chronis-Tuscano et al., 2010). Similarly, ADHD and anxiety have a co-occurrence rate nearing 50% (Kessler et al., 2006), and ADHD patients who presented with anxiety were more likely to struggle with emotion regulation (Reimherr et al., 2017).

Autism also has a high rate of co-occurrence with psychiatric disorders (Kirsch et al., 2020), with almost half of ASD individuals being diagnosed with at least two other conditions (Simonoff et al., 2008). Rates of co-occurrence with depression range anywhere from 20 to 70% (Simonoff et al., 2008). Importantly, ASD individuals who score higher on cognitive ability tests tend to have higher rates of depression and a lower sense of self-worth (Magnuson and Constantino, 2011). Social anxiety is another common co-occurring condition for ASD individuals, and is often correlated with decreased social skills, poor social competence, and low social motivation (Spain et al., 2018). Additionally, Rieske et al. (2013) found that severity of autism symptoms can account for approximately 50% of the variance in generalized anxiety symptoms.

Unfortunately, due to the lack of substantial research on non-diagnosed neurodivergent individuals, it is unclear how accurate these rates of co-occurrence are for the neurodivergent community as a whole. By excluding self-identified neurodivergent individuals from analyses on co-occuring conditions, the literature is lacking a clear understanding of how all neurodivergent individuals experience psychiatric conditions and how the experiences may differ depending on access to treatment or the affirming nature of a diagnosis.

### 1.4 Service utilization

Due to the high rate of co-occurring mental health issues and increased symptom severity, neurodivergent individuals have a high rate of mental health service utilization. Medically diagnosed ADHD and autistic individuals are more likely to utilize mental health care and psychiatric services compared to the general population, and spend almost three times as much on mental health care in a year (Zerbo et al., 2019). Additionally, diganosed ADHD or autistic adults are more likely to be taking antidepressant medication, or antipsychotic medication than the general public (Zerbo et al., 2019). This trend is similar in diagnosed neurodivergent children. Gupte-Singh et al. (2017) found that the average direct expenditure cost for ADHD children was almost twice as high as for non-ADHD children.

Despite the high rate of service utilization, neurodivergent individuals, regardless of diagnosis, still have lower quality of life, and academic and social outcomes (Bagwell et al., 2001; Barry et al., 2002; Van der Oord et al., 2005; Loe and Feldman, 2007; Fleming et al., 2017). These consistently poor outcomes indicate that existing services are not meeting the needs of neurodivergent individuals, either in quality or accessibility. A systematic review of the literature on care access for autistic families found major discrepancies in access to care, referral frequency, number of service hours, and proportion of unmet service needs based on socioeconomic status as well as racial and ethnic minority background (Smith et al., 2020). Moreover, once ASD individuals gain access to mental health care, they report a lack of therapist knowledge in treating autistic individuals (Adams and Young, 2021). National treatment data also suggests under-treatment of ADHD (Cuffe et al., 2009) and ADHD individuals often struggle with maintaining support as they transition out of childhood (Swift et al., 2014; Reale et al., 2018).

### 1.5 Neurodivergent affirming care

Improvements to neurodivergent affirming mental health care are cited as one of the most pressing needs for the neurodivergent community at present (Mason et al., 2023; Pantazakos and Vanaken, 2023). Importantly, neurodivergent affirming care is not based around the goal of “normalization” and does not require a medical diagnosis. While most historical treatment options for these conditions, such as ABA, focus on reducing neurodivergent symptoms and increasing “normal” behavior (Wilkenfield and McCarthy, 2020; Chapman and Bovell, 2022), affirming care aims to adapt common treatment methods so they are more effective with neurodivergent individuals in addressing underlying mental health concerns such as depression or anxiety. Studies have found that the wellbeing of neurodivergent individuals, and more specifically of autistic individuals, is dependent on perceived levels of support and acceptance from peers and family members (Renty and Roeyers, 2006; Milton and Sims, 2016; Cage et al., 2018; Di Renzo et al., 2020), and not in the reduction of neurodivergent symptoms directly.

While the neurodivergent community is still evolving, experts in the field have started to address the characteristics necessary for providing efficacious and ethical neurodivergent-affirming care. Simple adaptations have been suggested, such as discussing with clients how the treatment setting might be altered to reduce the activation of threat systems (lowering the lights, reducing aroma therapy, etc.), and asking clients what aspects of their neurodivergence they would like support in addressing (e.g., time-management, emotion regulation, etc.) as opposed to assuming all neurodivergent conditions need to be addressed (Jones et al., 2024). Compassion-focused therapy has been suggested as a way to balance dysregulated emotion regulation systems in neurodivergent individuals, and reduce feelings of shame and low-self compassion brought on by social stigma (Mason et al., 2023).

### 1.6 The present study

This study represents a quality improvement analysis within an intensive outpatient program that serves a high proportion of neurodivergent clients. This study has two main goals. The first goal is to examine the differences in symptoms at the time of intake between neurotypical clients, non-affirmed neurodivergent clients, and affirmed neurodivergent clients. The second goal is to assess the benefits of an affirming care model for all neurodivergent clients by analyzing symptom improvement at discharge. The hope is that these analyses will shed light on disparities in mental health symptoms between clients who have received affirming care and those who have not, and guide future treatment decisions when working with this population.

## 2 Materials and methods

### 2.1 Setting

Charlie Health is a virtual intensive outpatient program that treats clients between the ages of 10 and 34 who present with complex and highly acute mental health disorders. Programming at Charlie Health consists of 9 h of virtual group sessions per week, as well as 1 h of individual therapy and a 1 h family therapy session. Each group session is 3 h long, broken into 50 min sessions with 10 min breaks in between hours. These group sessions include 1 h of evidence-based, relationally-informed, guided process work; 1 h of experiential therapy (e.g., art, music, mindfulness) and; 1 h of evidence-based skill building curriculum. Group and individual sessions are offered throughout the day, 6 days a week, to increase accessibility. The average length of stay in the program is 10–12 weeks.

A key component of treatment at Charlie Health is a compassion-focused, identity-affirming, relationally-informed approach to care delivery. Each hour of an IOP session is adapted to create a neuro-inclusive, supportive environment for connecting around lived experiences. Curricular interventions address sensory needs, multiple dimensions of communication and expression, emotional and physical self care, and an exploration of needs for social and civic engagement. Varying support needs across contexts are explored and modification of environment, where possible, is encouraged to better accommodate individual needs. Across all offerings, affirming care is a central tenet when designing programming and support opportunities.

### 2.2 Ethics considerations

This study was reviewed and approved by the NorthStar Institutional Review Board (IRB) who deemed this investigation exempt as secondary research usage (NB400170).

### 2.3 Positionality statement

Drawing from their own experiences of navigating undiagnosed neurodivergent conditions for many years, the lead researcher of this study recognizes the critical importance of validation and tailored support for neurodivergent conditions in fostering resilience and recovery and aims to advocate from within the mental health care system. Additionally, their background is informed by previous quality improvement work with neurodivergent individuals. While they acknowledge their biases in favor of neurodivergent individuals, they are committed to assessing the data with humility and developing approaches to care that honor all unique identities and strengths. Furthermore, questions regarding neurodivergence were reviewed by Charlie Health’s director of clinical curriculum who identifies as neurodivergent. Finally, the analyses and report presented here were reviewed by multiple individuals who identify as neurodivergent, as well as individuals who do not identify as neurodivergent, but have a history of working with neurodivergent individuals.

### 2.4 Data collection

Between May 2023 and October 2023, intake data were collected from 6,753 participants. All data were self-reported. Clients were given an intake survey in the first hour of their orientation session. A Charlie Health staff member joined the group and distributed personalized links to each client, and then stayed to answer questions until all clients were finished. Clients were instructed that the survey was optional and would not affect their admission status. Of those 6,753 participants, 1,140 submitted discharge surveys at the time of the analysis. Discharge surveys were distributed on the client’s last day in group sessions. Clients were pulled into a breakout room on Zoom with a Charlie Health staff member who then gave the client a personalized link to their discharge survey. Clients were informed that the survey was optional and would not affect their discharge status. If the client opted out of the survey, they were sent back to their group; otherwise, once the survey was complete, they were sent back to their group. If a client missed their final group session, they were emailed and texted a personalized link to the discharge survey and prompted to fill it out with a $25 incentive.

### 2.5 Measures

#### 2.5.1 Demographics

Demographics were collected at intake. Clients were asked to disclose their age, gender (*male, female, non-binary, genderqueer, nonconforming, gender fluid, gender neutral*), sexual orientation (*straight, asexual or gray-sexual, bisexual, pansexual, gay, lesbian, queer, questioning*), race (*Black or African American, Indigenous peoples around the world, Asian, Middle Eastern or North African, White, other*), neurodivergent identity (*autism, attention deficit hyperactive disorder, dyslexia/dyscalculia, speech or language disorder, sensory processing disorder, tourettes, down syndrome, none, other*), and neurodivergent diagnosis (*autism, attention deficit hyperactive disorder, dyslexia/dyscalculia, speech or language disorder, sensory processing disorder, tourettes, down syndrome, none, I do not know, other*).

For this study a neurodivergent identity of “other” could be anything the client deemed neurodivergent due to the lack of a social definition for neurodivergence. The researchers aimed to emphasize that a client’s identity labels are their decision, and it is this act of identifying as neurodivergent that they aimed to analyze. All questions were multi-select to allow clients to select all answers that they felt represented them.

#### 2.5.2 Depression

The Patient Health Questionnaire Modified for Adolescents (PHQ-A) is a 9 item scale that was used to measure depressive symptoms. Questions are rated on a scale from 0 (“not at all”) to 3 (“nearly every day”). A sum score, ranging from 0 to 27 was calculated and scores of 5, 10, 15, and 20 represented mild, moderate, moderately severe, and severe depression, respectively (Johnson et al., 2002).

#### 2.5.3 Anxiety

The Generalized Anxiety Disorder-7 (GAD-7) is a 7 item scale that was used to measure anxiety symptoms. Questions are rated on a scale from 0 (“not at all”) to 3 (“nearly every day”). A sum score, ranging from 0 to 21 was calculated and score cut offs of 5, 10, and 15 represent mild, moderate, and severe anxiety, respectively (Spitzer et al., 2006).

#### 2.5.4 Self-harm

Self-harm was measured by asking clients how many days in the 30 days prior to the survey they had engaged in self-harm. Answers could range from 0 to 30.

### 2.6 Data preparation

To determine if a client had a fully affirming neurodivergent diagnosis, we reviewed our neurodivergent demographic questions and calculated a binary variable indicating whether a client’s reported neurodivergent identities matched their reported neurodivergent diagnoses. Additionally, we calculated binary variables to determine if clients had an ASD affirming diagnosis or an ADHD affirming diagnosis to further understand symptom differences within the more concise medical model. Only clients who completed treatment and were discharged on clinical recommendation from their individual therapist were included in the outcomes analyses.

### 2.7 Data analysis strategy

#### 2.7.1 Descriptive statistics

Descriptive statistics were run to gain a better understanding of the demographic distribution of the sample that was surveyed. This included age, gender, sexual orientation, race, neurodivergent identities, and neurodivergent diagnoses.

#### 2.7.2 Missing data

Missing data was analyzed using R Statistical Software (v4.4.1, R Core Team, 2021). Chi-square tests were run to compare missingness rates between demographic groups.

#### 2.7.3 Outcomes analyses

A series of one-way multivariate analysis of variance (MANOVA) tests were run to compare three groups–individuals who are neurotypical, who identify as neurodivergent and have an affirming diagnosis, and who identify as neurodivergent and do not have an affirming diagnosis– by the three outcome variables of depression, anxiety, and self-harm. Six sets of tests were run, including (1) neurodivergent groups predicting symptoms at intake, (2) neurodivergent groups predicting symptom change at discharge, (3) ADHD group predicting symptoms at intake, (4) ADHD group predicting symptom change at discharge, (5) Autism group predicting symptoms at intake, (6) Autism group predicting symptom change at discharge. All tests were run in SPSS version 29 (IBM Corp, 2020). When MANOVAs indicated significant differences, follow-up univariate one-way ANOVAs were conducted for each dependent variable. The assumptions of linearity, multicollinearity, outliers, multivariate normality, and adequate sample size were tested and met. The assumption of homogeneity was violated for the neurodivergent group’s symptoms at intake, and so the Games-Howell method was used for the post-hoc tests at intake. The assumption of homogeneity was met for the ADHD and Autism-specific groups, so a Tukey post-hoc test was used for intake symptoms within these two groups. In the ADHD and Autism-specific analyses, there were unequal sample sizes across groups, so Pillai’s Trace was used rather than Wilkes’ Lambda in the MANOVA tests.

## 3 Results

### 3.1 Demographics

Data were collected from 6,753 clients within Charlie Health’s IOP program who were in treatment between April 2023 and October 2023. Ages of this sample ranged from 10-years-old to 34 years-old, with a mean age of 18.66 years-old. 54.6% of clients fell within the adolescent (ADOL) age group (10–17), and 44.4% fell into the young adult (YA) population (18+). 51.9% of clients identified as female, 27.0% identified as male, and 11.7% identified as a gender minority group. 36.5% of clients identified as heterosexual or straight, and 51.9% of clients identified as a sexual minority. Table 1 details demographics further.

**Table 1:** Demographics collected at intake for all clients included in the analysis.

	Overall (*n* = 6,753)	Neurodivergent identity 61.4% (4,144/6753)	Neurodivergent diagnosis 56.6% (3,825/6753)
Age	18.66 (5.51)	18.96 (5.57)	18.86 (5.57)
Race			
Black or African American	10.0% (672/6753)	8.0% (330/4144)	7.7% (293/3825)
Indigenous People	2.3% (156/6753)	1.9% (78/4144)	1.9% (73/3825)
Asian	3.4% (230/6753)	2.8% (115/4144)	2.7% (102/3825)
Middle Eastern or North African	0.6% (39/6753)	0.5% (21/4144)	0.5% (18/3825)
White	64.1% (4,327/6753)	67.1% (2,782/4144)	67.5% (2,583/3825)
Other	7.1% (477/6753)	6.7% (276/4144)	6.6% (253/3825)
Missing	12.6% (852/6753)	13.1% (542/4144)	13.2% (503/3825)
Gender			
Female	51.9% (3,508/6753)	46.4% (1921/4144)	46.7% (1787/3825)
Male	27.0% (1820/6753)	26.3% (1,091/4144)	27.0% (1,034/3825)
Non-binary	5.3% (359/6753)	6.6% (274/4144)	6.2% (237/3825)
Genderqueer/non-conforming	1.1% (74/6753)	1.4% (57/4144)	1.3% (50/3825)
Gender fluid	2.4% (162/6753)	2.9% (119/4144)	2.9% (112/3825)
Gender questioning	1.1% (71/6753)	1.3% (52/4144)	1.1% (42/3825)
Gender neutral	0.3% (23/6753)	0.4% (15/4144)	0.4% (14/3825)
Other	1.5% (101/6753)	1.9% (77/4144)	1.7% (66/3825)
Missing	9.4% (635/6753)	13.0% (538/4144)	12.7% (483/3825)
Sexual orientation			
Asexual or gray-sexual	2.7% (181/6753)	2.8% (114/4144)	2.6% (100/3825)
Bisexual	20.3% (1,370/6753)	21.2% (879/4144)	21.0% (802/3825)
Pansexual	9.3% (630/6753)	11.5% (477/4144)	11.3% (434/3825)
Gay	2.4% (159/6753)	2.4% (101/4144)	2.4% (91/3825)
Heterosexual or straight	36.5% (2,462/6753)	29.5% (1,223/4144)	30.4% (1,163/3825)
Lesbian	3.6% (245/6753)	3.5% (147/4144)	3.6% (137/3825)
Queer	3.5% (235/6753)	4.4% (183/4144)	4.0% (153/3825)
Questioning	3.5% (236/6753)	3.5% (143/4144)	3.5% (134/3825)
Other	6.6% (449/6753)	6.6% (273/4144)	6.7% (258/3825)
Missing	11.6% (786/6753)	14.6% (604/4144)	14.5% (553/3825)

Table 2 demonstrates the breakdown of neurodivergent identities and diagnoses. Over half of the sample (61%) held at least 1 neurodivergent identity, and neurodivergent clients held an average of 1.4 neurodivergent identities, with ASD (17.0%) and ADHD (42.9%) being the most common. Slightly fewer clients endorsed having a neurodivergent medical diagnosis (56.6%). In total, 53.4% of neurodivergent identifying clients reported a diagnosis that fully affirmed their neurodivergent identity and approximately 8% of clients who reported a neurodivergent diagnosis did not identify as neurodivergent at all. When focusing on ASD and ADHD respectively, only 45.3% of clients who identified as being autistic had an autism diagnosis, and 75.5% of clients who identified as ADHD had an ADHD diagnosis.

**Table 2:** Neurodivergent identities and demographics collected at intake for all clients included in the analysis.

	Overall (*n* = 6,753)	Neurodivergent Identity 61.4% (4,144/6753)	Neurodivergent Diagnosis 56.6% (3,825/6753)
Neurodivergent identity			
Autism spectrum disorder (ASD)	17.0% (1,149/6753)	27.7% (1,149/4144)	26.1% (999/3825)
Attention deficit hyperactive disorder (ADHD)	42.9% (2,894/6753)	69.8% (2,894/4144)	67.8% (2,594/3825)
Dyslexia/Dyscalculia	8.7% (590/6753)	14.2% (590/4144)	13.5% (515/3825)
Language processing disorder	2.1% (145/6753)	3.5% (145/4144)	3.5% (132/3825)
Sensory processing disorder	7.0% (475/6753)	11.5% (475/4144)	11.0% (421/3825)
Tourettes	1.3% (90/6753)	2.2% (90/4144)	2.3% (87/3825)
Down syndrome	0.1% (9/6753)	0.2% (9/4144)	0.2% (7/3825)
Other	9.9% (669/6753)	16.1% (669/4144)	15.6% (597/3825)
Neurodivergent medical diagnosis			
Autism	8.2% (553/6753)	13.0% (540/4144)	14.5% (553/3825)
ADHD	34.3% (2,317/6753)	54.2% (2,244/4144)	60.5% (2,316/3825)
Dyslexia/Dyscalculia	5.0% (337/6753)	7.8% (324/4144)	8.8% (337/3825)
Language processing disorder	1.5% (99/6753)	2.1% (89/4144)	2.6% (99/3825)
Sensory processing disorder	2.8% (192/6753)	4.5% (187/4144)	5.0% (192/3825)
Tourettes	0.9% (63/6753)	1.5% (62/4144)	1.6% (63/3825)
Down syndrome	0.0% (1/6753)	0.0% (1/4144)	0.0% (1/3825)
Other	8.8% (593/6753)	13.1% (543/4144)	15.5% (592/3825)

### 3.2 Missing data

#### 3.2.1 Intake data

To better understand the patterns of missing data within our analyses, chi-square tests were run to assess missingness patterns on outcomes variables between clients who had a neurodivergent identity and those who did not. While there was a significant difference in the amount of missing PHQ data at intake between clients who identified as neurodivergent (5.43%) and those who did not (4.22%) (*p* = 0.0294), based on a chi-square test, the amount of overall missing data was only 4.96% and therefore we do not believe this difference will impact our outcomes. There was not a significant difference in the amount of missing GAD data or days of self-harm data at intake between neurodivergent identifying individuals and non-neurodivergent identifying individuals.

A similar missingness pattern emerged when reviewing the data for diagnosed neurodivergent clients compared to non-diagnosed clients. Based on a chi-squared test, diagnosed neurodivergent clients had significantly more missing PHQ9 data (5.65%) than those who did not report a neurodivergent diagnosis (4.06%) (*p* = 0.003), however since the total amount of missing PHQ9 data at intake is less than 5% we do not believe this will impact our findings. Furthermore, no significant differences in amounts of missing GAD data or days of self-harm data were found between diagnosed neurodivergent clients and non-diagnosed clients.

#### 3.2.2 Discharge data

Approximately 17% of clients who had intake surveys also submitted discharge surveys by the time of the analysis. A chi-square test showed no statistically significant difference in the rate of discharge survey submission between neurodivergent identifying individuals (16.24%) and neurotypical identifying individuals (17.90%) (*p* = 0.082). Furthermore, there was no difference in the patterns of missing PHQ9 data (*p* = 0.367), GAD7 data (*p* = 0.366) or days of self-harm data (*p* = 0.365).

Based on a chi-square test, individuals who did reported a neurodivergent diagnosis at intake were less likely to submit a discharge survey (15.89%) compared to individuals who did not report a neurodivergent identity (18.17%) (*p* = 0.015). This difference is small enough that we do not anticipate that it will affect our analyses. Diagnosed neurodivergent individuals were also more likely to have more missing PHQ9 data (74.77%) compared to non-diagnosed participants (72.43%) (*p* = 0.033). There were no significant differences in missingness of GAD7 or days of self-harm.

### 3.3 Broad neurodivergence

A MANOVA was run to determine the effect of neurodivergent identity and diagnosis on a client’s mental health symptoms at intake. Three measures of mental health symptoms were assessed: depression, generalized anxiety, and days of self-harm. Three groups of neurodivergent identity were included in the sample: neurotypical, neurodivergent without a fully affirming neurodivergent diagnosis (non-affirmed neurodivergent clients), and neurodivergent with a fully affirming neurodivergent diagnosis (affirmed neurodivergent clients). Average depression scores ranged from moderate to moderately severe, anxiety scores were in the moderate range, and days of self-harm were on the low end of clinically significant for all groups (Table 3). The differences between neurodivergent groups on combined dependent variables was statistically significant *F*(6, 12,134) = 27.204, *p* = <0.001, Wilks 𝝠 = 0.974; partial η^2^ = 0.013.

A follow-up univariate ANOVA showed that depression scores [*F*(2, 6,069) = 62.431, *p* < 0.001, partial η^2^ = 0.020], generalized anxiety scores [*F*(2, 6,069) = 73.649, *p* < 0.001, partial η^2^ = 0.024] and days of self-harm [*F*(2, 6,069) = 12.368, *p* < 0.001, partial η^2^ = 0.004] were all statistically significantly different depending on neurodivergent identity and diagnosis label. Games-Howell post-hoc tests were run for all dependent variables. Non-affirmed neurodivergent clients had significantly worse depression than neurotypical clients (*p* < 0.001) and affirmed neurodivergent clients (*p* < 0.001). Affirmed neurodivergent clients had significantly worse depression symptoms than neurotypical clients (*p* < 0.001). For anxiety, the post-hoc test showed that non-affirmed neurodivergent clients had significantly worse scores compared to neurotypical clients (*p* < 0.001), and affirmed neurodivergent clients (*p* < 0.001). Additionally, affirmed neurodivergent clients had significantly worse anxiety scores compared to neurotypical clients (*p* < 0.001). Finally, a third Game-Howell post-hoc test for self-harm days showed that non-affirmed neurodivergent clients reported significantly higher days of self-harm compared to neurotypical clients (*p* < 0.001), and affirmed neurodivergent clients (*p* = 0.003). However, there was no significant difference in the number of self-harm days at intake between neurotypical clients and affirmed neurodivergent clients (*p* = 0.297) (Table 3).

**Table 3:** Symptoms at intake by neurodivergent grouping.

Neurodivergent groups	Neurotypical		Non-affirmed ND		Affirmed ND		*F*(2,6,069)	η^2^
	*M*	*SD*	*M*	*SD*	*M*	*SD*		
Depression score at intake	13.1	7.2	15.5	6.7	14.1	7.2	62.431***	0.020
Anxiety score at intake	11.1	6.2	13.3	5.8	12.1	6.2	73.649***	0.024
Days of self-harm at intake	2.1	4.9	3.0	5.8	2.4	5.4	12.368***	0.004

***p < 0.001.

Another one-way MANOVA was run to identify any differences in change scores for mental health symptoms at discharge based on their neurodivergent identity and diagnosis group. Change scores, or deltas, from intake to discharge were calculated for depression scores, generalized anxiety scores, and days of self harm and were used as the dependent variables to measure change in mental health symptoms at discharge. The same three groups were assessed: neurotypical, neurodivergent without a fully affirming diagnosis, and neurodivergent with a fully affirming diagnosis (Table 4). The differences between groups of neurodivergent clients on combined dependent variables at discharge was not found to be statistically significant *F*(6, 1866) = 1.294, *p* = 0.256, Wilks 𝝠 = 0.992; partial η^2^ = 0.004.

**Table 4:** Symptoms at discharge by neurodivergent groupings.

Neurodivergent groups	Neurotypical		Non-affirmed neurotypical		Affirmed neurotypical	
	*M*	SD	*M*	SD	*M*	SD
Depression change score	7.7	6.9	8.1	6.2	7.7	7.0
Anxiety change score	6.4	5.6	7.0	5.8	6.0	6.0
Days of self-harm change score	1.8	5.0	2.4	5.2	1.6	5.2

### 3.4 ADHD and autism

To assess the effect of an affirming diagnosis and affirming care on a more tightly defined concept of neurodivergence, one-way MANOVAs were performed specifically looking at ADHD and Autism clients. Additional tests were conducted on clients with ADHD and autism identities due to sample size, and the frequency with which ADHD and autism are diagnosed in the larger population. Additional neurodivergent diagnoses captured in the data collection, such as dyslexia or Tourette’s, were not analyzed further because of the small sample sizes.

The MANOVA test for ADHD clients was run using depression scores, anxiety scores, and days of self-harm at intake as the dependent variables. The three groups of ADHD that were included were: Neurotypical clients, clients with an ADHD identity without an ADHD diagnosis (non-affirmed ADHD clients), and clients with an ADHD identity and an ADHD diagnosis (affirmed ADHD clients). Depression scores at intake once again ranged from moderate to moderately severe across all groups, anxiety scores fell within the moderate range, and days of self harm were on the low end of clinically significant (Table 5). The differences between groups of ADHD clients on combined variables was found to be statistically significant *F*(6, 10,180) = 18.207, *p* = <0.001, Pillai’s Trace = 0.021; partial η^2^ = 0.011.

**Table 5:** Symptoms at intake by ADHD groupings.

Measures	Neurotypical		Non-affirmed ADHD		Affirmed ADHD		*F*(2,5,091)	η^2^
	*M*	SD	*M*	SD	*M*	SD		
Depression	13.0	7.2	15.4	6.8	14.7	7.0	46.87***	0.018
Anxiety	11.1	6.2	13.2	5.9	12.6	6.1	48.899***	0.019
Days of self-harm	2.2	5.0	2.4	5.0	2.4	5.3	1.945	0.001

***p < 0.001.

To follow up, univariate ANOVAs showed that depression scores [*F*(2, 5,091) = 46.873, *p* < 0.001, partial η^2^ = 0.018], and anxiety scores [*F*(2, 5,091) = 48.899, p < 0.001, partial η^2^ = 0.019], were significantly different between the different groups of ADHD clients, while days of self-harm were not [*F*(2, 5,091) = 1.945, *p* = 0.143, partial η^2^ = 0.001]. A Tukey *post-hoc* test showed that non-affirmed ADHD clients had statistically significantly worse depression scores than neurotypical clients (*p* < 0.001), and affirmed ADHD clients (*p* = 0.045). Additionally, affirmed ADHD clients had significantly worse depression scores than Neurotypical clients (*p* < 0.001). For anxiety scores at intake, another Tukey *post-hoc* test was run and revealed that ADHD clients, regardless of an affirming diagnosis, had worse anxiety symptoms at intake than neurotypical clients (*p* < 0.001, *p* < 0.001). However, non-affirmed ADHD clients did not have significantly different anxiety scores than affirmed ADHD clients (*p* = 0.081). Finally, a Tukey *post-hoc* test revealed that there was no significant difference in the number of self-harm days between neurotypical clients and ADHD clients without an ADHD diagnosis (*p* = 0.431), or ADHD clients with an ADHD diagnosis (*p* = 0.153).

At discharge, change scores for depression, generalized, and days of self-harm were used to determine outcomes differences between the three groups of ADHD clients (Table 6). The differences between groups of ADHD clients at discharge on combined variables was not found to be statistically significant. *F*(6, 1,576) = 0.298, *p* = 0.938, Pillai’s Trace = 0.002; partial η^2^ = 0.001.

**Table 6:** Symptoms at discharge by ADHD groupings.

Neurodivergent groups	Neurotypical		Non-affirmed ADHD		Affirmed ADHD	
	*M*	SD	*M*	SD	*M*	SD
Depression change score	7.6	6.9	8.3	6.1	7.9	6.8
Anxiety change score	6.4	5.5	7.0	5.8	6.5	6.0
Days of self-harm change score	1.8	5.0	1.8	3.9	1.9	5.1

The same MANOVA tests were run to compare clients who identified as having ASD. Depression scores, anxiety scores, and self-harm days at intake were looked at as our dependent variables. The three groups of Autistic clients were: Neurotypical, Autistic identity without an Autism diagnosis (non-affirmed ASD), and Autistic identity with an Autism diagnosis (affirmed ASD). Average depression scores ranged from moderate to moderately severe, average anxiety scores were in the moderate range, and days of self-harm was at the low end of clinically significant (Table 7). The differences between groups of ASD clients on combined variables was found to be statistically significant *F*(6, 7,000) = 24.008, *p* = <0.001, Pillai’s Trace = 0.040; partial η^2^ = 0.020.

**Table 7:** Symptoms at intake by ASD groupings.

Measures	Neurotypical		Non-affirmed ASD		Affirmed ASD		*F*(2,3,501)	η^2^
	*M*	SD	*M*	SD	*M*	SD		
Depression	13.0	7.2	16.3	6.3	15.0	7.4	58.995***	0.033
Anxiety	11.1	6.2	13.9	5.4	12.8	6.5	59.292***	0.033
Days of self-harm	2.1	5.0	3.3	6.2	3.1	6.9	14.508***	0.008

***p < 0.001.

A univariate ANOVA was run as a follow up test and showed that depression scores [*F*(2, 3,501) = 58.995, *p* < 0.001, partial η^2^ = 0.033], anxiety scores [*F*(2, 3,501) = 59.292, *p* < 0.001, partial η^2^ = 0.033], and days of self-harm [*F*(2, 3,501) = 14.508, *p* < 0.001, partial η^2^ = 0.008] were statistically significantly different between the different ASD groups. A Tukey *post-hoc* test showed that non-affirmed ASD clients had significantly worse depressive scores than neurotypical clients (*p* < 0.001), and affirmed ASD clients (*p* = 0.006). Additionally, affirmed ASD clients also had significantly worse depressive scores than neurotypical clients (*p* < 0.001). Non-affirmed ASD clients had significantly worse anxiety scores than neurotypical clients (*p* < 0.001) and affirmed ASD clients (*p* = 0.005), while affirmed ASD clients had significantly worse anxiety scores than neurotypical clients (*p* < 0.001). Finally, non-affirmed ASD clients reported significantly more self-harm days at intake than neurotypical clients (*p* < 0.001), but not compared to affirmed ASD clients (*p* = 0.870). Affirmed ASD clients also reported significantly more days of self-harm than neurotypical clients (*p* < 0.001).

At discharge, depression score deltas, generalized anxiety score deltas, and days of self-harm deltas were used as our dependent variables to determine outcomes differences between the ASD groupings (Table 8). The differences between groups of ASD clients on combined variables was not found to be statistically significant at discharge *F*(6, 1,088) = 0.779, *p* = 0.586, Pillai’s Trace = 0.009; partial η^2^ = 0.004.

**Table 8:** Symptoms at discharge by ASD groupings.

Neurodivergent groups	Neurotypical		Non-affirmed ASD		Affirmed ASD	
	*M*	SD	*M*	SD	*M*	SD
Depression change score	7.6	6.9	7.7	5.9	7.0	7.1
Anxiety change score	6.4	5.5	6.3	5.5	5.9	6.1
Days of self-harm change score	1.8	5.1	2.8	5.6	1.5	4.2

## 4 Discussion

### 4.1 Importance of identity

This study provides clinical data that supports the need for neurodivergent-affirming care in mental health treatment for all clients who identify as neurodivergent, regardless of diagnosis. Within Charlie Health’s virtual intensive outpatient program, clients who identified as neurodivergent consistently reported higher levels of mental health symptoms at intake compared to their neurotypical counterparts. Additionally, non-affirmed neurodivergent clients reported worse mental health symptoms across the board compared to neurodivergent individuals who had received a fully affirming diagnosis. Non-affirmed clients scored worse in depression symptoms, anxiety symptoms, and days of self-harm compared to fully affirmed clients.

While the social definition of neurodivergence is still evolving, the medical definitions of neurodivergent conditions are much more rigid and therefore provide a more consistent response from clients. By narrowing the scope to only ADHD and autistic clients, we demonstrated that these patterns of mental health symptom presentation in affirmed and non-affirmed clients fit within the more tightly defined medical definition, as well as the broader social definition of neurodivergence. Clients who identified as being ADHD without the affirming ADHD diagnosis had significantly worse depression scores at intake compared to ADHD affirmed clients and neurotypical clients, and all ADHD clients regardless of affirming diagnosis had significantly worse anxiety scores than neurotypical clients. Additionally, non-affirmed autistic clients had significantly worse depression and anxiety scores compared to both neurotypical clients and fully-affirmed autistic clients, and all autistic clients regardless of diagnosis reported more days of self harm than neurotypical clients.

We found the differences in symptoms between our neurotypical and neurodivergent participants to be large enough to be clinically significant, confirming prior literature that showed lower quality of life for neurodivergent individuals (Barry et al., 2002; Van der Oord et al., 2005; Loe and Feldman, 2007; Fleming et al., 2017). The effect sizes for our affirmed and non-affirmed neurodivergent sample were smaller, however, they boarder on clinically significant. Because all participants are being treated for high acuity mental health conditions, we expected to see high rates of anxiety and depression across the board, making small variations in scores more practically significant. Variations in effect size between the general neurodivergent population, and our smaller sample of ASD and ADHD clients should also be noted. Effect sizes were larger when there was more flexibility in the social definition of neurodivergence. Further research examining depression and anxiety symptoms within affirmed and non-affirmed neurodivergent individuals who are not already high acuity will help us understand what these symptomology differences might look like outside of a high acuity population and how to address them in a therapy setting.

This study also demonstrates the effectiveness of affirming care for clients who identify as neurodivergent, regardless of diagnosis. Identity-affirming care does not need to come in the form of a diagnosis. The diagnosis of neurodevelopmental disorders is not a part of Charlie Health’s care model, yet clients who identified as neurodivergent without an affirming diagnosis had similar rates of improvement as those with an affirming diagnosis simply by participating in a program that worked to affirm and validate their lived experience while treating their mental health symptoms. The act of validating symptoms and experiences, allowing accommodations when requested, and exploring identity formation regardless of diagnosis, allowed all clients who identified as neurodivergent to benefit from treatment. As mentioned previously in this paper, these adaptations to treatment are just the start of what experts propose for neurodivergent affirming care (Jones et al., 2024).

These findings highlight the importance of acknowledging client identity and validating lived experience as a key component of mental health. When identity is not reflected in mental health care, neurodivergent clients suffer from more severe mental health symptoms, and ultimately access unhelpful services at an extremely high rate. By supporting a neurodivergent client’s identity, regardless of medical assessment or diagnosis, clients report strong outcomes post-treatment.

### 4.2 Constraints on generality

This study assessed anxiety and depression symptomology differences between neurotypical individuals, affirmed neurodivergent individuals, and non-affirmed neurodivergent individuals who were all receiving treatment for high acuity mental health concerns. As such, the generalizability of the results is limited to the population of youth and young adults who are experiencing high acuity mental health conditions and have the resources to receive care. Prior to further studies with a general sample, these results should not be used to predict mental health symptoms in the broader population. Additionally, as noted previously, the social definition of neurodivergence is in flux. As such, the findings in this paper may be constrained by the current social climate and understanding of what neurodivergence means and may be subject to change as the definition of neurodivergence changes.

### 4.3 Limitations and further research

The key limitation to the current study is the method of data collection. All data used for analysis was self-reported, which specifically posed challenges with verifying claims of neurodivergent diagnoses. Future research should aim to gather paperwork detailing any formal neurodevelopmental diagnoses from participants. This additional data collection method would provide a confirmation of diagnoses and allow for more verifiable data and therefore, more reliable analyses. Additionally, anxiety in this study was measured using a single standardized scale for generalized anxiety disorder. Patterns of anxiety presentation between diagnosed and undiagnosed neurodivergent individuals could be further improved by using multiple anxiety scales to determine differences between generalized anxiety, social anxiety, phobias, and more. Taking a closer look at how anxiety is presenting, and the common coping mechanisms used between each population may help us understand why rates of anxiety differ.

Further research is needed into what the agents of change are when receiving an affirming diagnosis. Theories include diagnoses affording additional resources, and feeling as though an official diagnosis lends support to identities that a client may still be unsure of. Additional research should also be conducted with the general population to assess how neurodivergent identities and affirming care affect mental health outside of a high acuity setting, therefore increasing the generalizability of these findings. Finally, as the social definition of neurodivergence changes, continued research is needed to track how these trends may change over time. Mixed methods studies may provide further insight into how and why these trends are changing.

## Data availability statement

The datasets presented in this article are not readily available because of the sensitive nature of the data. Requests to access the datasets should be directed to EK, elizabeth.kroll@charliehealth.com.

## Ethics statement

The studies involving humans were approved by Northstar Research Ethics Review Board. The studies were conducted in accordance with the local legislation and institutional requirements. Written informed consent for participation was not required from the participants or the participants’ legal guardians/next of kin because data was not identifiable and the project was deemed exempt under Common Rule 45 CFR 46.104(d)(4)(ii): Secondary research for which consent is not required.

## Author contributions

EK: Conceptualization, Data curation, Formal analysis, Investigation, Methodology, Project administration, Writing – original draft, Writing – review & editing. ML: Supervision, Writing – review & editing. JK: Conceptualization, Writing – review & editing. KK: Writing – review & editing. JB: Writing – review & editing. IZ: Writing – review & editing. CF: Supervision, Writing – review & editing.
